# Supplementary material for: Local Competition and Enhanced Defense: How Metarhizium brunneum Inhibits Verticillium longisporum in Oilseed Rape Plants
Source: J Fungi (Basel). 2023 Jul 28;9(8):796. doi: 10.3390/jof9080796 (PMC10455689; doi:10.3390/jof9080796)
Supplement: Supplementary file 1 [file jof-09-00796-s001.zip › jof-2451974-supplementary.pdf]

Supplementary material

Article title

Local Competition and Enhanced Defense: How *Metarhizium brunneum* Inhibits *Verticillium longisporum* in Oilseed Rape Plants

Catalina Posada-Vergara *, Stefan Vidal and Michael Rostás *

Agricultural Entomology, Department of Crop Sciences, University of Goettingen, Grisebachstr 6, 37077 Goettingen, Germany; svidal@gwdg.de

* Correspondence: cposada@gwdg.de (C.P.-V.); michael.rostas@uni-goettingen.de (M.R.)

Table S1. Primers used for qPCR amplification of genes of *Brassica napus* root tissues

ID	Gen/accession	Gene description	Sequence 5' --> 3'	Reference
**ABA2**	LOC106300040	Xanthoxin dehydrogenase	GCATCGCTCGTCTGTTCCAC	Karssemeijer et al., 2021
	XM_013736089.1		CGGCGAAGTCAACAGCGTTA	
**ERF2**	At5g47220	Ethylene Response Factor 2	ATGTACGGACAGAGCGAGGT	Yang et al., 2010
			AAGCTTCGAAACCAACAAGTAACTG	
**ACO**	EV102889	ACC oxidase	TCCGTCTGGGCTATCACTCT	Maag 2014
			GTGAGTGGGTCGATGTTCCT	
**PR1**	XM_013877950.1	Pathogenesis-related protein 1	AAAGCTACGCCGACCGACTACGAG	Alkooranee 2017
			CCAGAAAAGTCGGCGCTACTCCA	
**PAL**	LOC106342153 ?	phenylalanine ammonia-lyase 1	TCGCTATGGCTTCTTACTGCTCTG	Karssemeijer et al., 2021
	XM_013781008.1		GAGGTCTTACGAGATGAGATGAGTCC	
**AOS**	LOC106327419	Allene oxide synthase	ACCGCTTGCGACTAGGGATC	Karssemeijer et al., 2021
	XM_013765565.1		CAAAGTCCTTACCGGCGCAC	
**PDF1.2**	EV163328	Defensin-like protein 16	TCCATCACCCTTCTCTTTGC	Maag et al., 2014
			TTTTGGCACGCATAGTCGTA	
**ACTIN**	AF111812	Housekeeping gene	ATCGTCCTCAGTGGTGGTTC	Maag et al., 2014
			TTGATCTTCATGCTGCTTGG	
**CYP79B2**	At4G39950	CYTOCHROME P450, FAMILY 79, SUBFAMILY B, POLYPEPTIDE 2	AAGAGGTTGTGCTGCTCCG	Tytgat et al., 2013 Also in Marthur
			TCCAAGTGAAACCTTGAAGAAGTC	
**CYP83A1**	At4G13770	CYTOCHROME P450, FAMILY 83, SUBFAMILY A, POLYPEPTIDE 1	CTCCTTATCCCTCGTGCTTG	Mathur et al., 2013
			TGTCGTAACCAGCGATCTTG	
**ICS2Bn**	XM_022690315	Brassica napus isochorismate synthase 2, chloroplastic	GAATGATGCTCTTCCTCGCAGTT	This study
	LOC106431819		TCGGAGACAGAAACCTTCGGAT	

Table S2. Summary statistics of fungal colony area, and colony growth inhibition percentage of *Metarhizium brunneum* and *Verticillium longisporum* when growing in dual culture

	Df (1/8)			
**Fungus**	**dpi**	**F value**	**Pr (>F)**	Inhibition (% ± SE)
Metarhizium brunneum Cb15	14	1.5843	0.2436	
	17	3.6803	0.0913·	-8.48% ±1.68
	23	0.7325	0.417	
Verticillium longisporum Vl43	14	21.203	0.0017**	12.97% ±1.90
	17	13.419	0.0064**	19.75% ±2.05
	23	44.798	0.0002***	41.84% ±4.49

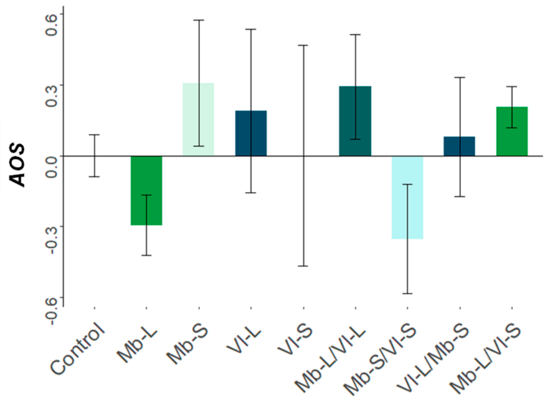

Figure S1. Expression of allene oxide synthase *AOS* gene
